# Emetophobia appears to be the most common specific phobia that requires treatment

**DOI:** 10.1192/bjo.2025.10947

**Published:** 2026-01-12

**Authors:** Adrian Meule

**Affiliations:** Department of Psychology, University of Regensburg, Regensburg, Germany

**Keywords:** Emetophobia, vomit phobia, fear of vomiting, phobia of vomiting, specific phobia

## Abstract

Emetophobia is a specific fear of vomiting. Although it is a relatively unknown anxiety disorder that has received limited attention in research, many psychotherapists are familiar with it because they frequently encounter persons with emetophobia in clinical practice. While animal-related phobias are the most common specific phobias in general, a recent study by Veale and colleagues (2025) suggests that, among persons seeking treatment for specific phobias, emetophobia appears to be the most prevalent. Furthermore, the study indicates that persons with emetophobia differ from those with other specific phobias (e.g. in terms of gender ratio or treatment setting). These findings dovetail with results from other recent studies suggesting that emetophobia may be a more impairing disorder and is, therefore, more likely to require treatment than other specific phobias.

**Figure:**
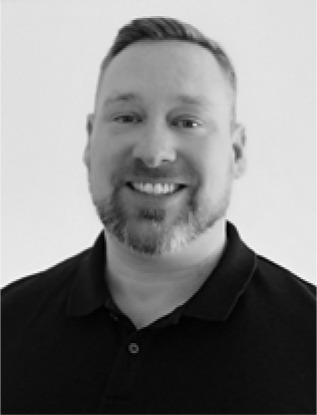

Emetophobia is a fear of vomiting that is classified as a specific phobia in current diagnostic systems.^1,2^ Most people with emetophobia fear that they, themselves, will have to vomit, or fear both vomiting and seeing others vomit.^3^ A minority of about 10–12% of persons with emetophobia exclusively fear seeing others vomit.^4^ As a result of these fears, persons with emetophobia show a wide range of both safety behaviours (e.g. checking expiry dates of food, hand-washing, checking own and others’ health status, overcooking food) and avoidance behaviours (e.g. refusing to go to school, avoiding sick or drunk people, avoiding consumption of certain foods or alcohol, avoiding travelling/public transportation^3^). There is also a high comorbidity with related conditions such as other anxiety disorders and obsessive–compulsive disorder,^4^ which may contribute to or reinforce these behaviours.

Earliest reports about individuals with emetophobia can be traced back to the middle of the 20th century. For example, Allen and Broster described a case of a young woman in 1945 who ‘vomited after eating sardines, at the age of 12, [and] developed an obsessional fear of this recurring’^5^. Other case reports describing children with a fear of vomiting were published in 1958 and 1961.^6,7^ While authors in the previous century used to describe this condition as either vomit phobia, phobia of vomiting or fear of vomiting, it seems that the term emetophobia was not used in the scientific literature until the 21st century.^8^ Although cases had already been described more than half a century ago, emetophobia has received little attention in research and is a relatively unknown disorder among the general public. However, it seems that many psychotherapists are familiar with it because they frequently encounter persons with emetophobia in clinical practice.^9^

Compared with other specific phobias (of which fear of animals is the most common), emetophobia is among the least common.^10^ However, a recent study by Veale and colleagues now suggests that it may, in fact, be the most common specific phobia that requires treatment.^11^ Specifically, the authors analysed the data of more than 1000 UK patients who had received treatment for a specific phobia and found that around 20% presented with emetophobia, which was the most prevalent specific phobia subtype in this sample. Moreover, patients with emetophobia differed from those with other specific phobias in that the former were more likely to be female and to have received in-patient treatment. They also tended to be younger than those with other specific phobias, although this was found only for adults.

Veale and colleagues’ study is a valuable contribution to the literature on emetophobia because there have been, in fact, only a handful of studies that examined possible differences between emetophobia and other specific phobias.^10,12,13^ Importantly, the study suggests that, although emetophobia is a relatively rare condition, the numerous safety and avoidance behaviours seem to impair daily functioning much more than do other specific phobias, which is why it more often requires treatment (and even more intensive in-patient treatment) than other specific phobias.

This concurs well with a recent study that retrospectively analysed the data of 110 persons with specific phobia as primary diagnosis and who had received in-patient treatment at the Schoen Clinic Roseneck (Prien am Chiemsee, Germany) between January 2015 and February 2024.^13^ Of these individuals, 70 had emetophobia and 40 had other specific phobia subtypes (e.g. fear of going to school, test anxiety, phagophobia, blood/injection/injury-type phobia). These numbers are interesting in themselves because they suggest that, if persons with a specific phobia receive in-patient treatment (which is a fairly uncommon treatment setting for this condition), those with emetophobia are over-represented in such a sample compared with the overall population of those with specific phobias, in line with the findings by Veale and colleagues.^11^ This may be partially explained by the fact that persons with emetophobia often restrict food intake, with many presenting as underweight and, thus, require a more intensive treatment setting.^13,14^

Figure 1 depicts differences between in-patients with emetophobia and those with other specific phobias. As can be seen, the former were younger (a finding that is also in line with Veale and colleagues’ study), had a lower body weight and reported both lower life satisfaction and higher phobic anxiety. In contrast to Veale and colleagues’ study, gender distribution did not differ between groups in this study. However, the female:male ratio of 9:1 in adults reported by Veale and colleagues^11^ is in accord with a recent meta-analysis that produced a pooled estimate, across 21 samples, of 91% of (mostly adult) persons with emetophobia being female.^4^

**Fig. 1:**
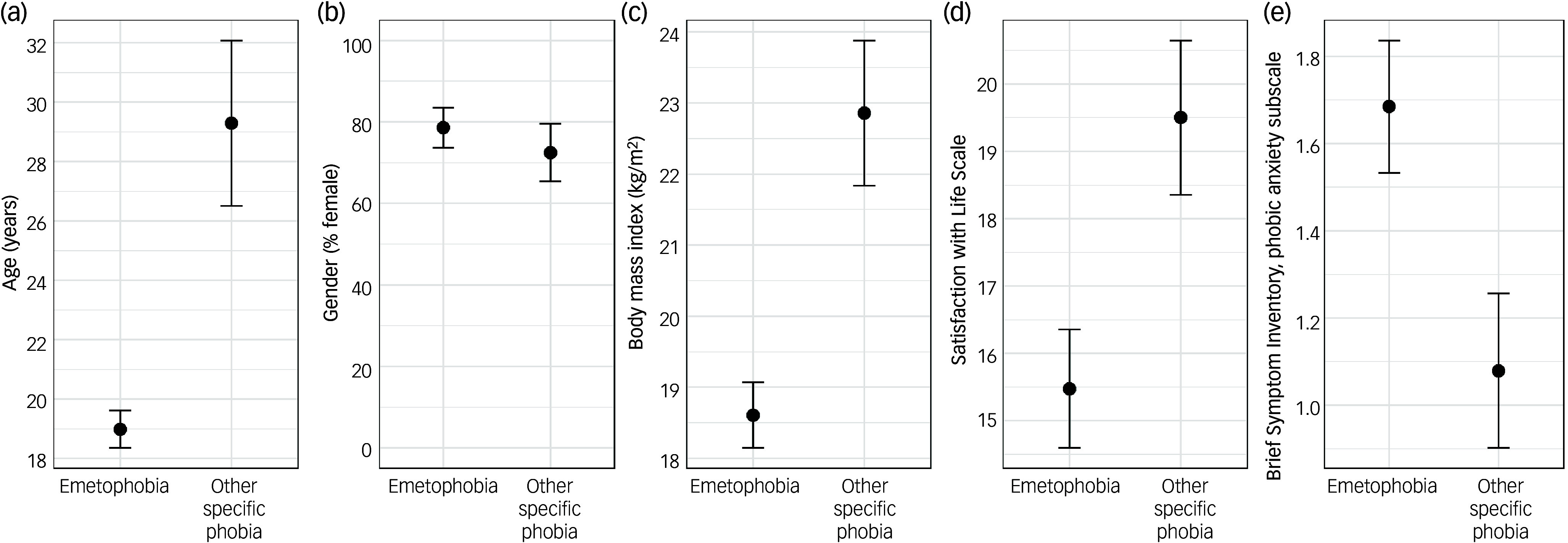
(a) Mean age, (b) percentage of females, (c) mean body mass index, (d) mean sum scores on the Satisfaction with Life Scale and (e) mean scores on the phobic anxiety subscale of the Brief Symptom Inventory in persons with emetophobia and those with other specific phobias at admission to in-patient treatment. Error bars indicate s.e.m. This figure is based on the data reported in ref. ^13^

In conclusion, emetophobia is a rare condition that, nonetheless, appears to be one of the most commonly treated specific phobias. Although it can usually be treated in an out-patient setting,^15^ its impairing impact on daily functioning sometimes necessitates more intensive in-patient treatment. While early reports of emetophobia date back to the mid-20th century, research has only recently begun systematically to examine its distinct characteristics. Given its debilitating nature and unique clinical presentation, further research is essential to refine treatment approaches and raise awareness among both healthcare professionals and the general public.

## References

[1] American Psychiatric Association. Diagnostic and Statistical Manual of Mental Disorders 5th ed. American Psychiatric Association, 2013.

[2] World Health Organization. International Classification of Diseases 11th ed. World Health Organization, 2022.

[3] Keyes A , Gilpin HR , Veale D. Phenomenology, epidemiology, co-morbidity and treatment of a specific phobia of vomiting: a systematic review of an understudied disorder. Clin Psychol Rev 2018; 60: 15–31. 29277320 10.1016/j.cpr.2017.12.002

[4] Meule A , Seufert L , Kolar DR. Emetophobia (fear of vomiting): a meta-analysis. J Anxiety Disord 2025; 114: 103053. 40700922 10.1016/j.janxdis.2025.103053

[5] Allen C , Broster LR. Paranoid psychosis successfully treated by adrenalectomy. Br Med J 1945; 1: 696–8. 20786074 10.1136/bmj.1.4402.696 PMC2062422

[6] Sutton HA , Falstein EI , Judas I. Emotional reactions to medical procedures and illness in a hospital child psychiatry unit. Am J Orthopsychiatry 1958; 28: 180–7. 13498125 10.1111/j.1939-0025.1958.tb03735.x

[7] Blitzer JR , Rollins N , Blackwell A. Children who starve themselves: anorexia nervosa. Psychosom Med 1961; 23: 369–83. 13870017 10.1097/00006842-196109000-00001

[8] Lipsitz JD , Fyer AJ , Paterniti A , Klein DF. Emetophobia: preliminary results of an internet survey. Depress Anxiety 2001; 14: 149–52. 11668669 10.1002/da.1058

[9] Vandereycken W. Media hype, diagnostic fad or genuine disorder? Professionals’ opinions about night eating syndrome, orthorexia, muscle dysmorphia, and emetophobia. Eat Disord 2011; 19: 145–55. 21360365 10.1080/10640266.2011.551634

[10] Becker ES , Rinck M , Türke V , Kause P , Goodwin R , Neumer S , et al. Epidemiology of specific phobia subtypes: findings from the Dresden Mental Health Study. Eur Psychiatry 2007; 22: 69–74. 17157482 10.1016/j.eurpsy.2006.09.006

[11] Veale D , Beeson C , Papageorgiou A. Frequency of and sex distribution in specific phobia subtypes in a treatment-seeking sample. BJPsych Open 2025; 11: e164. 40744468 10.1192/bjo.2025.10767 PMC12344425

[12] Davidson AL , Boyle C , Lauchlan F. Scared to lose control? General and health locus of control in females with a phobia of vomiting. J Clin Psychol 2008; 64: 30–9. 18161045 10.1002/jclp.20431

[13] Meule A , Zisler EM , Metzner MS , Voderholzer U , Kolar DR. Characteristics of and treatment outcome in inpatients with emetophobia and other specific phobias. J Psychiatr Res 2025; 189: 285–90. 40543409 10.1016/j.jpsychires.2025.06.028

[14] Veale D , Costa A , Murphy P , Ellison N. Abnormal eating behaviour in people with a specific phobia of vomiting (emetophobia). Eur Eat Disord Rev 2012; 20: 414–8. 22081507 10.1002/erv.1159

[15] Boschen MJ , Jones K. A clinician’s quick guide to evidence-based approaches: emetophobia (specific phobia of vomiting). Clin Psychol 2024; 28: 75–8.

